# Vacuum brazing of Al_2_O_3_ and 3D printed Ti6Al4V lap-joints using high entropy driven AlZnCuFeSi filler

**DOI:** 10.1038/s41598-021-87705-x

**Published:** 2021-04-30

**Authors:** Ashutosh Sharma, Byungmin Ahn

**Affiliations:** 1 Department of Materials Science and Engineering, Ajou University, Suwon, 16499 Korea; 2 Department of Energy Systems Research, Ajou University, Suwon, 16499 Korea

**Keywords:** Materials for devices, Structural materials

## Abstract

In this work, we studied the brazing characteristics of Al_2_O_3_ and 3D printed Ti–6Al–4V alloys using a novel equiatomic AlZnCuFeSi high entropy alloy filler (HEAF). The HEAF was prepared by mechanical alloying of the constituent powder and spark plasma sintering (SPS) approach. The filler microstructure, wettability and melting point were investigated. The mechanical and joint strength properties were also evaluated. The results showed that the developed AlZnCuFeSi HEAF consists of a dual phase (Cu–Zn, face-centered cubic (FCC)) and Al–Fe–Si rich (base centered cubic, BCC) phases. The phase structure of the (Cu–Al + Ti–Fe–Si)/solid solution promises a robust joint between Al_2_O_3_ and Ti–6Al–4V. In addition, the joint interfacial reaction was found to be modulated by the brazing temperature and time because of the altered activity of Ti and Zn. The optimum shear strength reached 84 MPa when the joint was brazed at 1050 °C for 60 s. The results can be promising for the integration of completely different materials using the entropy driven fillers developed in this study.

## Introduction

Ceramic–metal joining is a hotspot for research because of the various applications such as heat exchangers, connectors, capacitors, thermoelectric, solar cells, and complex structural joints^1–3^. The most common and widely used ceramic is alumina, which has interesting properties such as high melting point, good hardness, excellent chemical resistance, and high wear/corrosion resistance. With the advent of modern engineering devices and technologies, more applications require highly pure alumina and composite ceramics. In many cases, alumina needs to be bonded to metal to serve certain functions^4–6^. The popular techniques used for this purpose over the decades include active metal brazing, diffusion bonding, ultrasonic bonding, microwave joining, and solid-state bonding (with and without interlayers). The role of wettability during metal/ceramic joining has been studied by several researchers, and it has been observed that the key property that leads to the success of a metal/ceramic joint is the ability of the filler to wet, spread, and react chemically with the faying surface^7,8^. However, the task of bonding a ceramic with metal is challenging owing to their wide differences in physicochemical and mechanical properties^2^.

Various active metal brazing fillers have been developed, with the most popular ones being eutectic AgCu, AgCuTi, AgCuSnTi, AgCuInTi, and Ti^9–13^. The eutectic composition of the Ag-28Cu (M.P. ≈ 780 °C) system is often preferred to limit the interface thermal stresses and maintain ductility. However, most of these active fillers contain expensive silver metal which makes the materials costly.

Other advanced brazing fillers contain micro- and nanoparticles as reinforcement in the filler matrix to promote wetting and joint strength^7,8,14–16^. The last decade has experienced immense advances in nanoparticle-reinforced solder and brazing alloys as a potential technology for controlling the wetting and refining the grains of intermetallic compounds (IMCs) in studies related to low-temperature Al-and Sn-brazing, and the resulting materials have shown remarkably improved wettability and joint performance. However, the use of nanoparticles has always been criticized for joining applications because of the inherent nanoparticle segregation and induced dispersion impurities^16^. The presence of large amounts of brittle IMCs at the joint interface is deteriorating for the performance of brazed implants. Therefore, brazing fillers with almost no segregation and impurities should be used for the joining of biomedical implants (Ti–6Al–4V) and abutment (ceramic) joints.

Recently, high-entropy alloys (HEAs) have shifted the alloy design approach to a new paradigm. HEAs have novel core effects, such as high mixing entropy, sluggish diffusion (less chance of formation of IMCs), solid-solution phases, and lattice distortion. Various HEAs have been designed so far in various fields such as aerospace, automobiles, energy storage, sensing, and nuclear plants^17,18^. Despite these attractive developments of HEAs for various applications, their roles in microjoining are still lacking.

For a high-entropy effect, the Gibbs free energy of mixing (ΔG_mix_) is more negative, and hence, the individual elements are preferably distributed in a muddled fashion instead of forming IMCs^19,20^. There are various methods to prepare the HEAs in literature. Most of the previous reports are based on arc melting and casting methods^17–20^. Powder metallurgy is becoming popular worldwide due to its benefits like uniform microstructure and extended solid solubility through mechanical alloying. Additionally, sintered products are free from shrinkage solidification defects which are usually prominent in casting methods. Recently, there are few notable works appeared on 3D printing of HEA and multi-material joining^21–24^. However, additive manufacturing process has few obstacles in its commercial use. For example, the process has interdependent process parameters, and usually difficult to control for obtaining a better microstructural characteristic. Besides, formation of acicular martensite structures in 3D printed HEA can be harmful to the joint characteristics which are absent in HEAs produced via SPS process. Control of martensite structures further requires annealing process to restrict their acicular shape to equiaxed grains. Therefore, in this study, we have developed an AlZnCuFeSi alloy using advanced powder metallurgy techniques. A non-Cantor dual BCC + FCC type microstructure was obtained to balance the ductility and strength of the HEAF to suit the Al-, Fe-, and Cu-components. Si and Fe provide high-temperature stability and machinability, respectively. Zn provides wetting of the metallic components for flux-free brazing^25^. The effects of the brazing temperature and time on the interface structure and joint strength were studied, and the mechanism of joining was reported.

## Materials and methods

### High entropy alloy filler

The constituent elemental powders: Al, Cu, Si, Zn, and Fe (purity > 99%, > 325 mesh, Sigma-Aldrich, USA) were used. High energy ball milling (RETSCH, RM-400, Germany) was used to achieve complete homogeneity of powder mixture. High energy balling has been shown to produce ultrafine grained and homogeneous alloys with extended solid solubility as compared to traditional powder blenders^26,27^. The milling conditions (milling time, ball to powder weight ratio (BPR), disk and vial rotation speeds in rpm, process control agent (PCA)) and high entropy parameters (enthalpy of mixing (ΔH_Mix_), mixing entropy (ΔS_Mix_), valence electron concentration (VEC), atomic size mismatch (δ), Pauling’s electronegativity (Δχ), interaction parameter (Ω), etc.) are listed in Table 1. Consolidation of the obtained brazing filler was performed via an SPS machine (Dr. Sinter 515S, SPS Syntex, Japan) at 650 °C, 100 °C/min, and 500 MPa in an Ar atmosphere.

**Table 1:** Milling conditions of HEAF powders.

HEAF milling conditions						
Composition (at.%)		Milling time (h)	BPR	Vial (rpm)	Disk (rpm)	PCA
Al_20_Cu_20_Si_20_Zn_20_Fe_20_		45	10:1	600	300	Stearic acid (0.2 wt.%)
**Calculated parameters**						
ΔH_Mix_ (kJ·mol^−1^)	ΔS_Mix_ (J·K^−1^·mol^−1^)	Phase	VEC	δ (%)	Δχ	Ω
− 13.76	13.38	BCC + FCC	7.6	7.16	0.135	1.26

### Joint materials and brazing conditions

**Figure 1:**
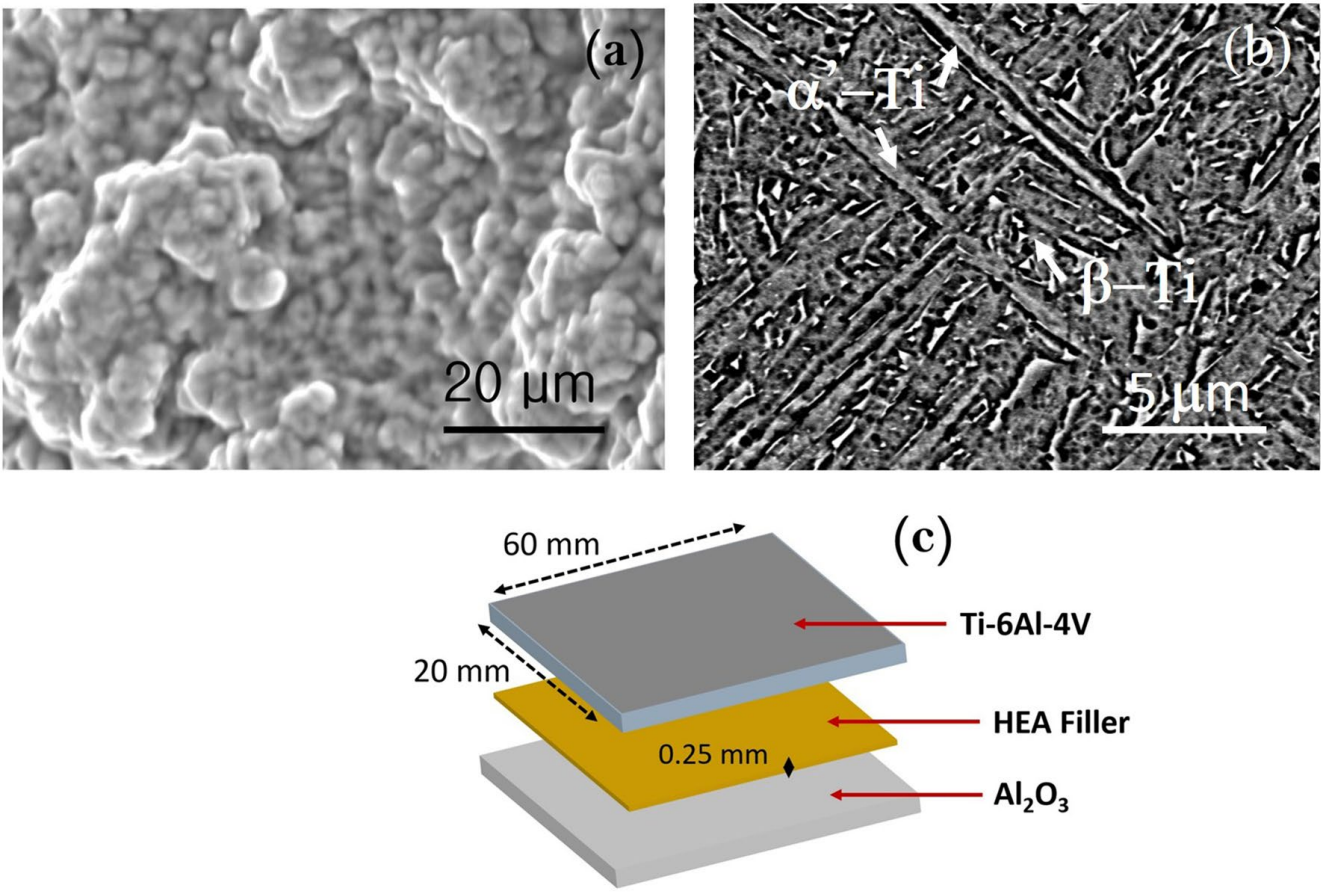
Microstructures of joint materials; (**a**) Al_2_O_3_ (92%) and (**b**) Ti–6Al–4V (3D printed), and (**c**) schematic of joint components.

Al_2_O_3_ was a sintered block procured from Sinoshine Industrial Ceramic Co., Ltd., China (92% pure, Model: SS-AWT-92). Ti–6Al–4V was additive manufactured by HS HI-TECH, Korea using an EOS M290 selective laser melting machine. The plate shape of Ti–6Al–4V was 3D printed using a fiber laser in argon atmosphere at a laser scan speed of 1250 mm/s. The laser power was 200 W. The layer thickness and hatch spacing were 80 and 100 µm respectively. The distribution of Ti–6Al–4V powder alloy was 45–75 µm. The initial microstructures of Al_2_O_3_ and Ti–6Al–4V are shown in Fig. 1a,b. The Ti–6Al–4V contains the α’-Ti (acicular) and β-Ti phases. Table 2 lists the composition of the joint materials and brazing conditions. The joint was made in the face-to face configuration, as shown in Fig. 1c.

**Table 2:** Joint materials and brazing conditions.

Components	Values
Al_2_O_3_ (wt%)	92% Al_2_O_3_, 4% SiO_2_, 3% MnO_2_, 1% MgO
3D printed Ti–6Al–4V (wt%)	90% Ti, 6% Al, 4% V
Brazing temperature (°C)	990, 1010, 1030, 1050, 1070, 1090
Brazing time (s)	20, 40, 60, 80, 100, 120
Atmosphere	Vacuum (3 × 10^–5^ Torr)

### Structural analysis

All the samples were characterized using a Rigaku Benchtop X-ray diffractometer (XRD). The powder crystallite size and lattice strain were estimated using the Williamson–Hall equation^28^. The dislocation density and phase fraction were estimated by the variance range method given by Sanchez and Cumbrera^29^. According to the integral breadth method, the size strain broadening caused by the crystallite size and lattice strains is expressed as:

1 $${\beta }_{i}=\frac{A}{{I}_{0}}$$

where A = area of the peak and I_0_ is the height of observed line profile. The peak broadening and anisotropy in the strain is caused by the dislocations in the crystal. The strain induced contribution to integral breadth (${\beta }_{i})$ can be expressed as (in s units, $s=\frac{2sin\theta }{\lambda }$ due to the dislocation arrangement in the crystal)^30^,

2 $${{(\beta }_{s})}_{i}^{D}=2\upvarepsilon s=4\upvarepsilon \frac{sin\theta }{\lambda }$$

The integral breadth in units of “s” can be now equated as:

3 $${{(\beta }_{2\theta })}_{i}^{D}=\frac{\lambda }{cos\theta }{{(\beta }_{s})}_{i}^{D}$$

Substituting (2) in (3)

4 $${{(\beta }_{2\theta })}_{i}^{D}=\frac{\lambda }{cos\theta }4\upvarepsilon \frac{sin\theta }{\lambda }=4\upvarepsilon \frac{sin\theta }{cos\theta }=4\upepsilon\tan\theta$$

Now according to Scherrer equation, the broadening due to crystallite size alone is

5 $${{(\beta }_{2\theta })}_{i}^{D}= \frac{K\lambda }{Dcos\theta }$$

or,

6 $${{(\beta }_{s})}_{i}^{D}= \frac{K}{D}$$

According to the Voight method, the integral breadth (${\beta }_{i})$ can be shown as the sum of size (D) and strain contributions (S):

7 $${\beta }_{i}={\beta }_{SC}+{\beta }_{DC}(\mathrm{Cauchy}/\mathrm{Cauchy})$$

8 $${{\beta }_{i}}^{2}={\left({\beta }_{SG}\right)}^{2}+{\left({\beta }_{DG}\right)}^{2} (\mathrm{Gaussian}/\mathrm{Gaussian})$$

Here, C and G represents respective Cauchy and Gaussian terms. Substituting Eqs. (2) and (6) in (7),

9 $${\left({\beta }_{s}\right)}_{i}= \frac{1}{D}+4\upvarepsilon sin\theta (\mathrm{Cauchy}/\mathrm{Cauchy})$$

Similarly, substituting Eqs. (2) and (6) in (8),

10 $${\left[{\left({\beta }_{s}\right)}_{i}\right]}^{2}={\left[\frac{1}{D}\right]}^{2}+{16{\upvarepsilon }^{2}\left[\frac{sin\theta }{\lambda }\right]}^{2}(\mathrm{Gaussian}/\mathrm{Gaussian})$$

From Eq. (3),

11 $${\left({\beta }_{s}\right)}_{i}=\frac{cos\theta }{\lambda }{\left({\beta }_{2\theta }\right)}_{i}$$

Substituting this in Eq. (9) and (10), and assuming a Gaussian distribution for strain (ε) and Cauchy distribution for crystallite size (D), we have:

12 $${\left[\frac{cos\theta }{\lambda }{\left({\beta }_{2\theta }\right)}_{i}\right]}^{2}=\frac{1}{D}+{16{\upvarepsilon }^{2}\left[\frac{sin\theta }{\lambda }\right]}^{2}$$

After Haider and Wagner, the parabolic approximation for the integral breadth of the Voigt function is given by^30^:

13 $$\frac{{\beta }_{C}}{\beta }=1-{\left(\frac{{\beta }_{G}}{\beta }\right)}^{2}$$

Substituting the values in Eqs. (4) and (5), and for simplicity, we put ${\left({\beta }_{2\theta }\right)}_{i}$ =$\beta$

14 $$\frac{K\lambda }{\beta Dcos\theta }=1-\frac{16{\upvarepsilon }^{2}}{{\beta }^{2}{cot}^{2}\theta }$$

After rearranging above equation,

15 $${\beta^2}/tan\theta \, = \, K\lambda /D* \, \beta /tan\theta sin\theta \, + \, 16 \, {\varepsilon^2}$$

or,

16 $${\beta^2}/tan\theta \, = \, 0.9\lambda /D* \, \beta si{n^2}\theta /cos\theta \, + \, 16 \, {\varepsilon^2}$$

Here, β is the integral breadth, and θ corresponds to the Bragg angle. The shape factor K = 0.9. The crystallite size of the given phase was determined by the linear plot of β^2^/tanθ against β/tanθsinθ measured from the respective major peaks in the XRD pattern. The crystallite sizes (D) and strain (ε) were determined from the slope and intercept at the ordinate axis. The phase fraction of a given phase was estimated using Eq. (17) given by Kumar et al.^31^.

17 $${X}_{k}= \left(\frac{{I}_{k}^{{h}_{k}, {k}_{k}, {l}_{k}}}{\sum {I}_{k}^{{h}_{k}, {k}_{k}, {l}_{k}}}\right)$$

where ${X}_{k}$ is the volume fraction of the *k*th phase, and ${I}_{k}^{{h}_{k}, {k}_{k}, {l}_{k}}$ is the integrated intensity of the (*h*_*k*_*, k*_*k*_*, l*_*k*_) plane. The dislocation density (ρ) can be calculated as follows^29^:

18 $$\rho =\frac{3.464 {<{\varepsilon }^{2}>}^{1/2} }{D*b}$$

Here, *b* and D are Burger’ vector and crystallite size for the respective phases.

### Microstructural analysis

The morphology of the samples was examined by field emission scanning electron microscopy (FESEM, FEI, Nova NanoSEM 450, USA) equipped with an energy dispersive X-ray spectroscopy (EDS) and a transmission electron microscope (TEM, FEI Tecnai G20 twin, USA). The SPSed samples were cut into dimensions of 10 mm × 10 mm with a diamond saw. The samples were mounted with a hot press and then polished to a 3 µm diamond paste. For TEM observations, the samples were prepared by a dual-beam focused Ga ion beam system (FIB/3D Analytical FIB-SEM NX 9000, Hitachi, Japan) with 1 mm^2^ square samples at 5 kV at an angle of 4°.

The experimental density of the bulk samples was estimated using Archimedes’ law in deionized water. The theoretical density of the sintered samples was determined from the density of the individual elements by applying the rule of mixtures. According to Mridha et al.^32^, the relative sintered density (w) of the compacts was estimated from the ratio of the measured (ρ_i_) and theoretical density (ρ_t_):

19 $$\mathrm{w }= \left(\frac{{\rho }_{i}}{{\rho }_{t}}\right)\times 100\%$$

### Melting behavior evaluation

Differential thermal analysis (DTA) analysis was carried out for the 45 h milled sample to determine the melting point of the alloy using the DTA analyzer (TG/DTA Simultaneous Measuring Instrument, Shimadzu, Japan) at a heating rate of 10 °C from 50 to 1200 °C under a nitrogen atmosphere using alumina pans.

### Microhardness measurement

The Vickers microhardness was measured using a microhardness testing machine (Mitutoyo HM-500, Japan) at a load of 100 gf for 15 s. Ten measurements were performed for each sample at random locations on the polished compacts. The microhardness was tested across the cross-section over all phases present and finally averaged values were reported as the final microhardness of the samples.

### Brazeability assessment

**Figure 2:**
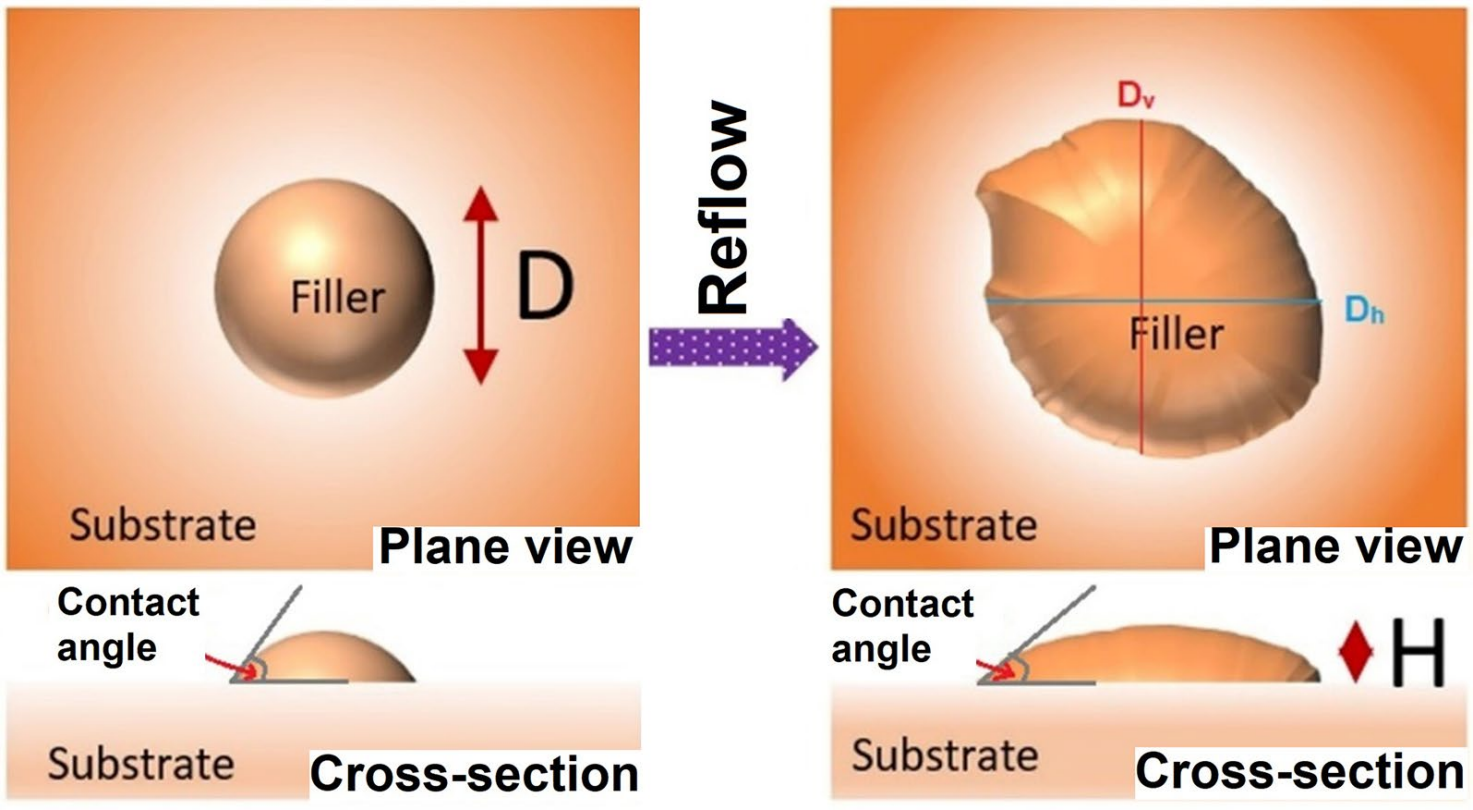
Schematic diagram for evaluation of filler brazeability.

The brazeability of the HEAs was assessed by Japanese standards given by JIS 3197 (2012). The HEA powder (0.2 g) milled for 45 h was placed over the substrates (Al_2_O_3_, Ti, Cu, and Fe) and heated to 990 °C and 1050 °C for 30 min under an Ar atmosphere and cooled to room temperature. After melting and alloy solidification, the spreadability was calculated using the following equation^33^, as shown in Fig. 2.

20 $$S= \left(\frac{D-H}{D}\right)\times 100$$

Here, D is the diameter of the filler spread, and the filler is assumed to be a perfect sphere (D = 1.24 ρ^1/3^). ρ is the mass per unit density of the filler, and H is the height of the filler spread after spreading. Both plane and cross-section schematics of the filler spread on the substrate are shown (Fig. 2). For a non-uniform spread area, the equivalent diameter may generally be calculated by the vertical (D_v_) and horizontal diameters (D_h_). This calculated diameter is often called as Feret’s diameter, which is related to the equivalent diameter according to the following equation^34^:

21 $$D \, = \, {D_{eq}} = \, {\left( {{D^2}_h{D_v}} \right)^{1/3}}$$

The contact angle was also determined by the cross-section images captured by a low magnification microscope as shown schematically in Fig. 2.

### Brazed joint interface and joint strength evaluation

**Figure 3:**
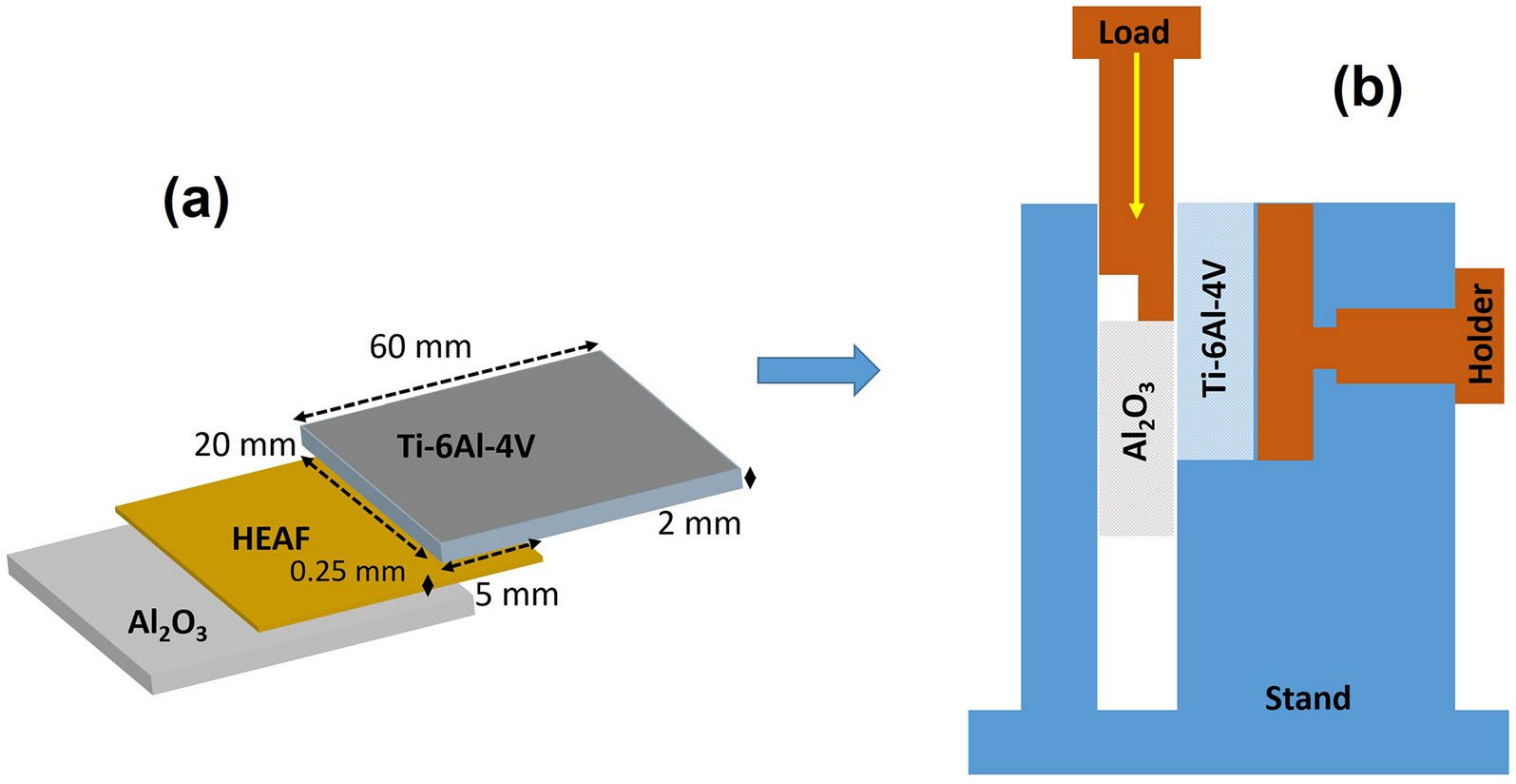
(**a**) Joint structure for (**b**) shear test evaluation.

The sizes of the Al_2_O_3_ and Ti–6Al–4V specimens used for brazing were 60 × 20 × 2 mm^3^, as shown in Fig. 3a. The brazing was performed at 990–1070 °C at 10 °C/min in a vacuum furnace of 3 × 10^−5^ Torr in a face-to-face joint, as shown in Fig. 3a. The holding time was also varied from 20 to 120 s. A small pressure of 5 × 10^–3^ Torr was applied on the Al_2_O_3_ face during brazing. Shear tests were performed in the lap joint configuration, as shown in Fig. 3b, using a universal testing machine (UTM) (DUT 30,000 CN, Korea). The tests were performed at room temperature at a crosshead speed of 5 mm/min.

## Results and discussion

### Filler synthesis and phase evolution

**Figure 4:**
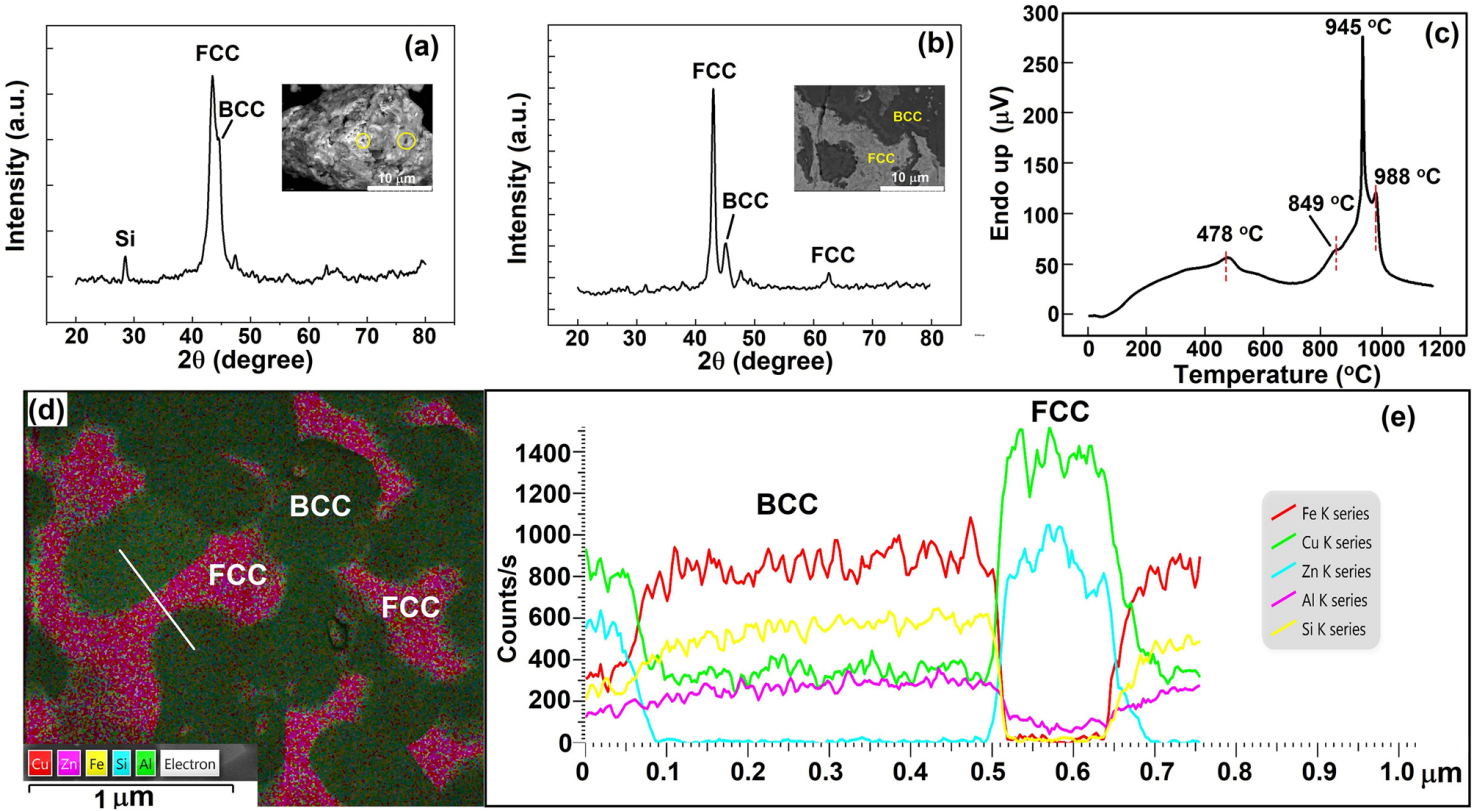
(**a**) XRD patterns of the filler powder after 45 h of milling and (**b**) densified filler after SPS at 650 °C. The inset shows the SEM images corresponding to each (**a**,**b**). (**c**) melting point of the HEAF in this study, (**d**) TEM-EDS mapping images of densified HEA, and (**e**) line profile analysis across the region in (**d**).

Figure 4a shows the XRD patterns of the filler powder after 45 h of milling. An FCC phase HEA is evident with a trace peak of Si. The black spots show the Si particles in the filler powder. A minor BCC phase was also observed. The presence of Si may be due to its strong covalent bonds and the hard diamond cubic structure compared to other constituent elements^35^. The crystallite size after high energy ball milling was found to be ≈82 nm for samples milled for 45 h. After densification at 650 °C, the XRD pattern showed a complete distinct dual-phase FCC + BCC structure (Fig. 4b). The distinct black and gray regions are marked with their compositions (Fig. 4b; inset).

Knowledge of the melting point is desirable for brazing. The DTA scan over the temperature range of 50–1200 °C is shown in Fig. 4c. It can be observed that the baseline shifted prominently during the initial stages, which may be related to various thermal transients. The small peak around 478 °C showed the evaporation of Zn in the initial stages. A sigmoidal behavior was observed where two endothermic peaks were obtained between 470 °C and 850 °C. Previous studies have indicated that a sigmoid curve represents the melting of the alternative zones between soft FCC and hard BCC-rich regions during cooling^36^. However, there was no evidence of other IMCs or laves phases because of the rapid cooling during SPS sintering. The shifting was also observed at higher temperatures, and a small peak manifesting a phase change was noticed at 988 °C, indicating the melting of the Al–Fe–Si phase. The amount of heat evolved was not significant for this peak, indicating a diffusional transformation without any sharp melting temperatures. The melting temperature of this alloy was thus 945 °C. Notably, the alloy was produced by milling, and the powder had a lot of residual stress associated. Thus, the thermal shift may have occurred as a result of the stress-released procedure at high temperatures^37^.

The FCC phase consists of approximately 61–64at% Cu and 32–33at% Zn. This composition is similar to FCC-brass alloy. The BCC and FCC phase fractions were 0.16 and 0.84 after 45 h of milling, which changed to 0.33 and 0.67 after SPS. The EDS line scan analysis shown in Fig. 4d-e confirms the formation of dual-phase HEAF.

Table 3 shows the various physicochemical properties of the constituent elements of the HEA^38,39^. This shows that Cu possesses a positive mixing enthalpy, leading to its poor miscibility over other elements, and favors the evolution of the FCC Cu–Zn phase. As reported by Pradeep et al.^40^, the BCC phase segregates as Cu–Zn in AlCrCuFeNiZn after sintering at 600 °C. In our study, a similar behavior was observed for the AlCuSiZnFe HEAs.

**Table 3:** Enthalpy of mixing (kJ/mol) of binary elements^31,32,35,38,39^.

Elements	Al	Si	Cu	Zn	Fe
Al	Al	− 19.0	− 1.0	− 1.0	− 11.0
Si		Si	− 19	− 18	− 35.0
Cu			Cu	1.0	13.0
Zn				Zn	4.0
Fe					Fe

### Filler density and hardness

The filler properties, density, and hardness are listed in Table 4. The sintered density of the samples was estimated to be 98% because of the rapid diffusion of the powder particles. The measured density is close to various Ti-based alloys (5.24 g/cm^3^) which seems beneficial for Ti–6Al–4V brazing. The microhardness of the densified filler was 875 VHN (Table 4). Besides, the dislocation density in bulk HEAF (≈ 5.8 × 10^16^ m^−2^) decreases down as compared to high energy ball milled filler powder (≈ 12.1 × 10^16^ m^−2^). This may be due to the release of stresses after high-temperature SPS at 650 °C. The FCC and BCC phase fractions in bulk HEAF were 0.67 and 0.33. The yield strength (YS) and the microhardness of the HEAF were related through the following equation^41^:

22 $$YS \, \left( {MPa} \right) \, = 3 \, \times \, {H_V}\left( {VHN} \right)$$

**Table 4:** Filler density and hardness.

Powder filler crystallite size	80 nm after 45 h
Lattice strain	1.66%
Powder filler dislocation density	12.1 × (10^16^ m^−2^) after 45 h
Sintered filler dislocation density	5.8 × (10^16^ m^−2^) at 650 °C
Sintered filler phase fraction	FCC: 0.67, BCC: 0.33
Sintered density	98%
Measured density	5.24 g/cc
Microhardness	875 Hv

Hence, the YS from Eq. (11) can be estimated to be 2625 MPa for AlCuSiZnFe HEA, which is close to that of Inconel 718, 625^42^. Such a high hardness value can be attributed to the finer grains and solid-solution hardening because of the duplex Cu–Zn and Fe–Si-rich microstructure^43,44^.

### Filler brazeability

**Figure 5:**
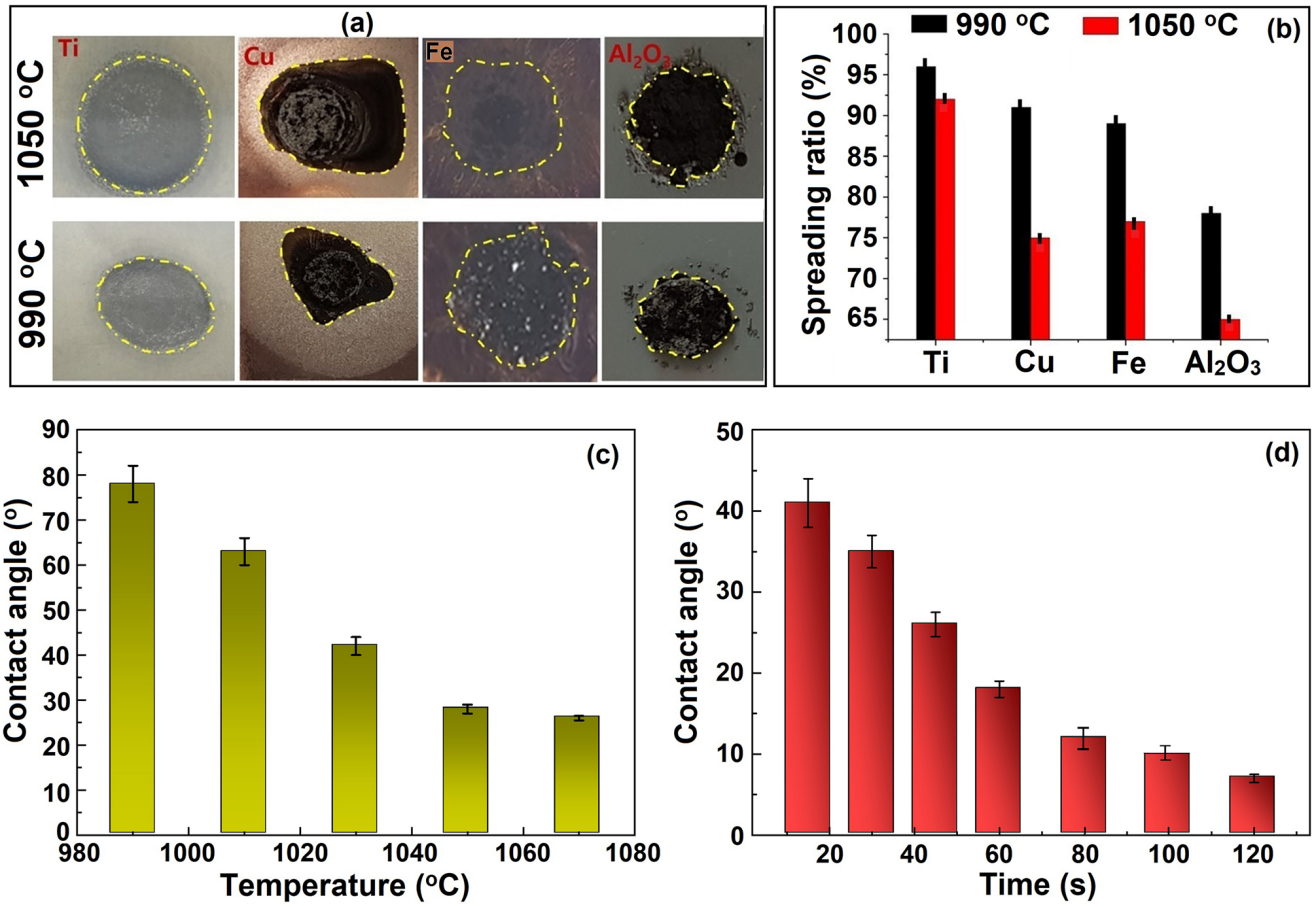
(**a**) Spread ratio images at different temperatures and substrates; (**b**) calculated spread ratio; (**c**,**d**) contact angles measured at different temperatures and times on Ti–6Al–4V.

Figure 5a shows the spreading images of the HEA on various contact substrates. In addition to Al_2_O_3_, we also chose Fe, Ti, and Cu for comparison. The spreading tests were performed at various temperatures without any flux. All the substrates showed a marked variation in the spreading behavior of the filler. The measured spreading ratios of the different samples are shown in Fig. 5b. The spreading ratio (S) was the maximum in the Ti sample followed by Cu, Fe, and Al_2_O_3._ The spreading ratio decreased in Fe and Cu slightly as compared to Ti because of the progressive oxidation at high temperature, and the substrate turned black. It was shown that the spreading of the HEA at 990 °C was approximately 78% for alumina, 89% for Fe, and 91% for Cu, which reached a maximum of 96% for Ti. The spread ratio further decreased to 92% on Ti, 75% on Cu, 77% on Fe, and 65% on Al_2_O_3_ at 1050 °C. This was attributed to the close association between pure Cu–Zn–Al–Fe and Ti. In other words, when most of the Zn is consumed, the active Ti cannot participate in the reactive wetting of alumina^45^.

Therefore, S decreases when the temperature exceeds 990 °C, and the wettability increases at a slow rate beyond 1050 °C. Although S was considerably smaller on alumina, it slightly decreased on the copper substrates. This may be because of the inertness of the ceramic and strong covalent bonding compared to metallic bonding in metals^11,46^. Muolo et al. also observed similar behavior while joining of refractory ceramics to Ti–6Al–4V alloy^46,47^.

The contact angle changes with the brazing temperature and times (Fig. 5c,d). Initally, at 990 °C, the contact angle was very high at approximately 78°, indicating the wetting of alumina. When the temperature increased, the contact angle gradually decreased and reached a minimum at 1070 °C. The change in contact angle at 1050 °C was observed up to 120 s (Fig. 5d). The contact angle decreased gradually to 18° at 60 s and maintained a stable value of 10° beyond 80 s. Compared to the sluggish wetting of Ag-, Pd-, or Au-based filler alloys on ceramics^47–49^, the present results revealed a better wettability of HEAF. Mulolo et al. reported an unsatisfactory wetting of ZrB_2_ ceramic when Ag, Cu and Ag–Cu fillers were used. The wetting was enhanced only after addition of active element (Zr) to Ag alloy^47^. In another research, Chen et al. reported that the addition of Cr as an active element in PdNi alloy promoted the brazing C–C composite^41^. Similar results were reported by He et al. for the joining of SiC ceramics with Au–Ni–Pd–Ti brazing alloy^42^. Our results are comparable to those of expensive brazing filler alloys such as Ag-, Au- and Pd-based alloys as reported in Ref^47–49^. This advantage is due to the instantaneous melting and homogeneous structure of the high-entropy alloy, which accelerates the diffusion and surface reaction during the high brazing process. In addition, too low contact angle beyond 60 s may not be sufficient to hold the lap-joints in position due to the excessive fluidity of the filler. Based on these observations, a brazing temperature of 1050 °C for 60 s can be promising for a good performance in the wettability tests on metal and ceramic substrates. This observation suggests that the developed HEAF can be used to join two completely different materials such as ceramics to metal joining.

### Effect of brazing temperature

**Figure 6:**
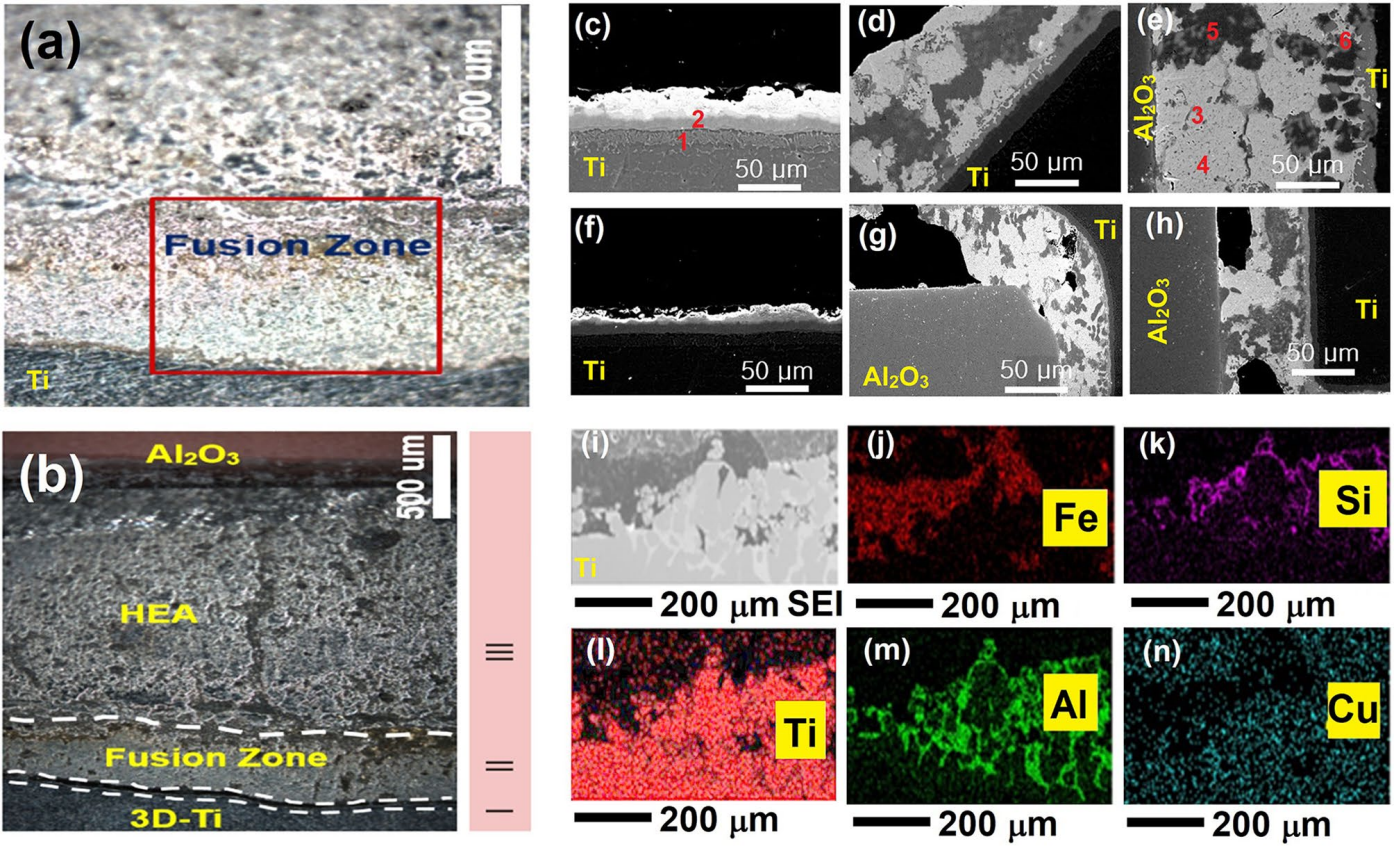
Brazed joint microstructure (**a**) 1050 °C, 60 s, (**b**) magnified view of (**a**). At (**c**–**e**) various temperatures (1010, 1050, and 1070 °C; time is fixed to 60 s) and (**f**–**h**) times (30, 60, and 120 s; temperature is fixed to 1050 °C). The EDS maps (**i**–**n**) were recorded for (**a**).

Figure 6 presents the joint interface between Al_2_O_3_ and Ti brazed with the developed HEAF. Figure 6a,b shows the low-magnification joint interface cross-section. We can see that the interface can be divided into three sections; I, II, and III. Section I and II consist of fine layers of Ti- and Cu-rich compounds near the Ti side. Various bright and dark networks of different phases were observed in section III. The broad braze seam (section III) is investigated in Fig. 6c-e at various temperatures. It can be seen that the variation in temperature considerably affects the interface characteristics. Initially, at a lower brazing temperature of 1010 °C, the wettability of the filler was very small, and the fusion zone thickness was also very small (approximately 43 μm) toward the Ti side. A fine Ti–rich layer existed along the alumina side owing to its dissolution from Ti–6Al–4V. The diffusion of filler into the ceramic leads to its reaction with the oxide-forming aggregate spots in the alumina substrate^9,11,46,50^. These aggregate spots may further improve the joining and strength. This is expected due to the higher wettability of Ti than that of alumina^6^. Owing to the high-entropy effect, all the elements were distributed inside the brazing seam. At a higher brazing temperature of 1050 °C, high entropy still occurs, and the dissolution of Al and Ti occurs into the brazing seam, which further stabilizes the Ti–Fe–Si phase. The thickness of fusion zone thickness also improved to 112 μm. When the brazing temperature approaches 1070 °C, Al can react with Ti or Cu without priority and continuously (the mixing enthalpy of Al–Ti is − 40.481 kJ/mol, Ti–Si − 66 kJ/mol, Al–Cu: − 1 kJ/mol)^50,51^. The EDS analysis showed that the reaction zone changes into two layers; Ti–Al and Ti–Cu compounds (Section I and II) and the diffusion zone is larger (176 μm). The braze seam also grows because of the formation of CuAl_2_ IMCs in section III. Considerable growth of IMCs is observed, and the interface becomes weaker. The activity of Ti is restrained because of the sufficient amount of Al and the lowering of Zn content because of its evaporation.

### Effect of brazing time

The joint characteristics at 1050 °C for different holding times are shown in Fig. 6f-h. It can be seen that the effect of brazing time is smaller than the effect of the brazing temperature. The reaction zone grows with higher brazing times. The HEAF diffuses into the alumina part owing to the presence of open pores on the surface. Because the alumina is 92% pure, there are some open pores caused by minor impurities. In addition, the diffusivity can also be attributed to the mechanism of capillary action of the filler on the alumina side of the joint^9,11,46^. Across the Ti side, as discussed the interface consists of Ti–Fe–Si between the filler metal and Ti and indicates Ti-rich and Si-rich constituents, respectively. The interfacial fusion zone thickness varies from 24, 53 and 79 µm at 30, 60 and 120 s, respectively. Figure 6i–n shows the electron probe microanalysis (EPMA) elemental maps of the interfacial region. The diffusion of Ti from the Ti surface and Cu from the filler metal across interface forms various Ti–Fe–Si and Cu–Ti, forming a stronger metal bond at the interface. The compositions of compounds 1–6 in Fig. 6c-n are given in Table 5.

**Table 5:** EDS results (in at.%) on both sides of the joint materials in Fig. 6.

Sites	Ti	Al	Si	Cu	Zn	Fe	V	Possible phases
1	26.6	32.5	8.1	7.7	12.7	9.7	2.7	Ti–Al
2	25.2	9.7	5.9	36.0	8.5	10.9	3.8	Ti–Cu
3	78.1	1.3	17.1	0.4	1.6	0.7	0.8	Ti_5_Si_3_
4	3.1	18.6	12.6	34.1	26.7	2.5	2.4	Cu–Zn
5	27.9	6.3	26.9	6.2	0.3	28.9	3.5	Ti–Fe–Si
6	0.8	54.2	2.4	36.4	3.6	0.4	2.2	Cu–Al

The atomic ratios of Ti and Si were approximately 5:3 in different regions. Moreover, Al was detected, and Fe and Si were the most prominent across the interface. The other element (Zn) was present in trace amounts. Ti was predominantly distributed in the form of Ti–Fe–Si and Ti–Al compounds closer to the Ti surface.

### Diffusion kinetics and growth

Considering alumina side, we assume no diffusion from the Al occurs from the alumina substrate. The migration of Ti atoms occurs from the Ti–6Al–4V substrate across the joint towards alumina. The binary phase diagrams of Ti and constituent elements show the formation of new phases across Ti–6Al–4V/HEA side owing to a mutual diffusion process. According to previous reports, the calculation of diffusion parameters of Ti using Fick’s second law might be difficult in such case^52,53^. Thus, we assume that the high entropy layer growth follows a parabolic power law according to the following equation:

23 $$X= {k}_{p}{t}^{n}={k}_{0}{t}^{n}{e}^{-\frac{Q}{RT}}$$

where *t* = brazing time, T = brazing temperature, *k*_*p*_ = penetration coefficient, *k*_*o*_ = constant depending upon IMC growth, Q = activation energy for diffusion, and R = gas constant.

**Figure 7:**
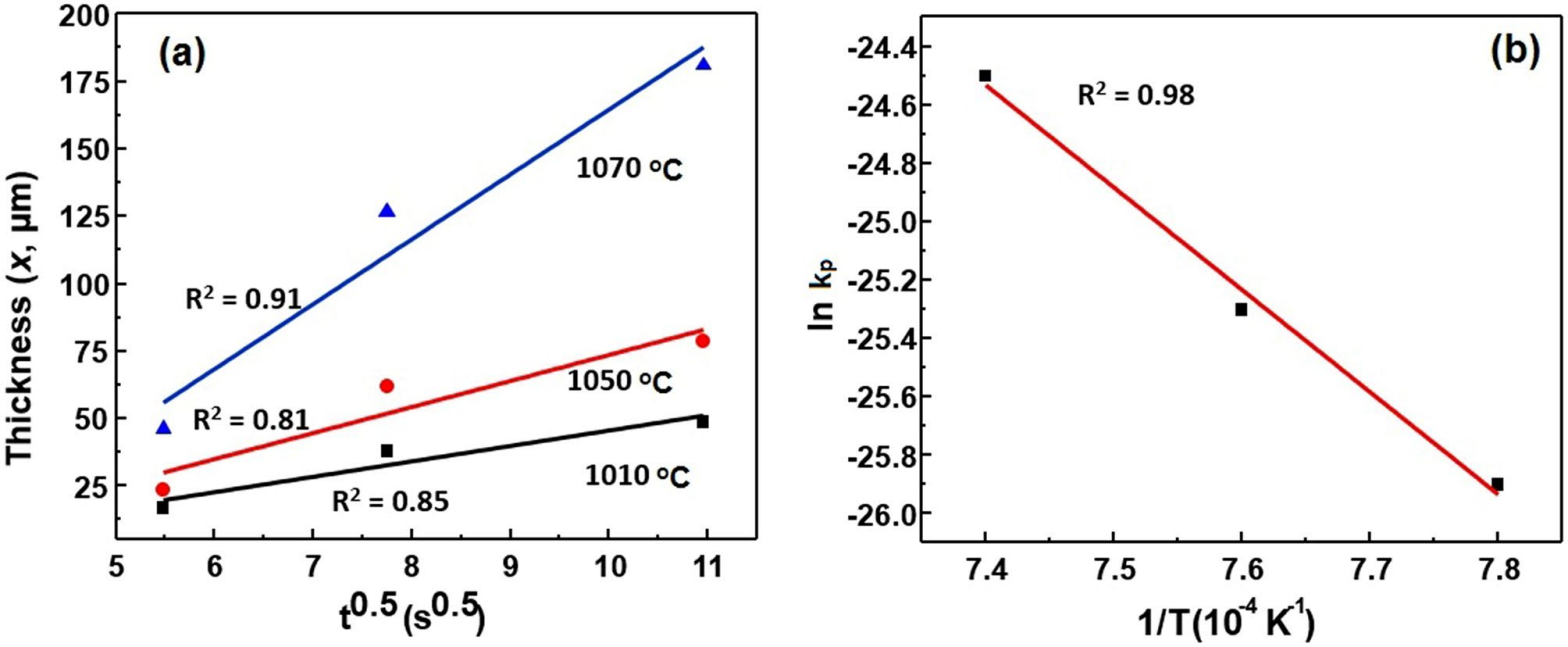
(**a**) The thickness of high entropy interfacial layer at various brazing conditions, and (**b**) Arrhenius plot of lnk_p_ vs 1/T.

Figure 7 shows the observed high entropy layer thickness for Al_2_O_3_/Ti–6Al–4V joints plotted with square root of the brazing time. The reaction layer grows more rapidly across the Ti–6Al–4V side with brazing temperature. Also, at prolonged brazing time, the growth rate increases at a slower speed (Fig. 7a,b). It is inferred that the elemental diffusion is reduced greatly either by a concentration-dependent diffusivity or through impingement by the formation of IMCs Ti_5_Si_3_ (Table 5). Taking logarithm of both sides of Eq. (23) and assuming *n* = 0.5,

24 $$lnX=\frac{-Q}{2RT}+\frac{1}{2}ln({k}_{0}t)$$

and,

25 $$lnX=\frac{1}{2}ln(t)+\frac{1}{2}ln(k_p)$$

The results show that Q = 291,007 J/mol and *k*_*0*_ = 0.58 × 10^–11^ m^2^/s from the Arrhenius plot. The Eqs. (24) and (25) show that the diffusion temperature and time affects the thickness of reaction layer and can be balanced by choosing a suitable brazing parameter (1050 °C, 60 s) for Ti–6Al–4V/HEA couple.

### Joint mechanism

**Figure 8:**
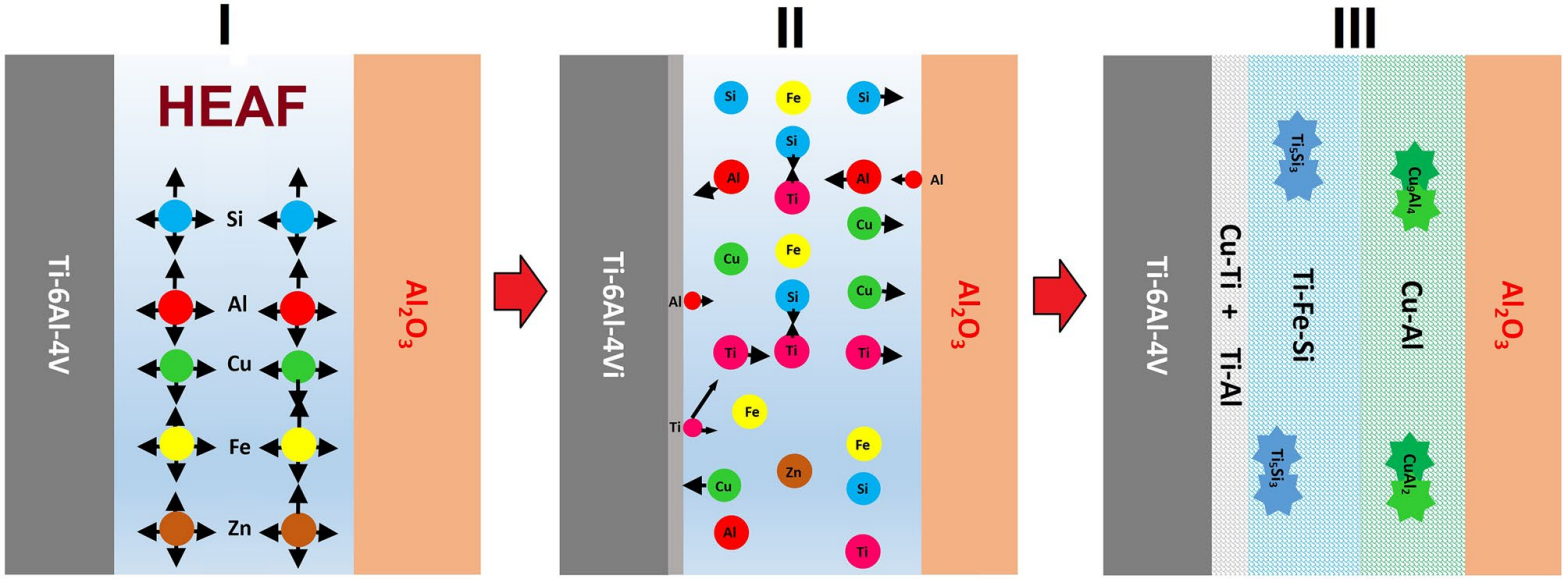
Microstructural model for the Al_2_O_3_/Ti–6Al–4V joints at various stages.

To understand the evolution mechanism of the Al_2_O_3_/Ti–6Al–4V joint, a schematic model is devised in Fig. 8. The brazed joint can be divided into three stages. The melting point of the AlCuSiZnFe filler was 945 °C. During the first stage, when the temperature exceeded 990 °C, the Ti/AlCuSiZnFe filler region softened and melting occurred. All the individual elements were mixed with one another because of the eutectic coupling of Ti and other constituent elements. First, Ti can easily react with Si to form IMCs because of the negative enthalpy of mixing (− 66 kJ/mol) in conventional alloys^32,35,50,51^. However, for the Ti/AlCuSiZnFe composite system, a random solid solution is formed because of the high entropy. It is also noteworthy that the mixing enthalpy of Fe and Si (− 35 kJ/mol) is highly negative compared to that of Fe and Al (− 11 kJ/mol) or between Ti and Si (− 66 kJ/mol). As a result, the formation of Ti_5_Si_3_ is more probable than that of Fe–Si IMCs.

The active element Ti dissolves into the AlCuSiZnFe alloy or diffuses to the alumina side as soon as the temperature reaches 1050 °C during the second stage. Similarly, the active element Zn also diffuses to the alumina side, and Zn, being the least melted component, is evaporated the most in the process. The third stage involves the migration of various compounds formed across the brazed seam. A spot of Cu–Al diffuses and forms CuAl_2_ IMCs near alumina at 1070 °C. CuAl_2_ has the lowest enthalpy of formation among the possible IMCs (CuAl_2_: − 6.1 kJ/mol, CuAl: − 5.1 kJ/mol, and Cu_9_Al_4_: − 4.1 kJ/mol) as reported by Xu et al.^54^. In addition, Si and Fe atoms form participates and are associated with the brazing seam. The IMCs formed CuAl_2_, and Ti_5_Si_3_ promoted solution hardening of the matrix phase, as observed by Tseng et al.^50^.

Consequently, the Cu–Al and Ti–Al layers become thicker with time, and the contents of Si and Fe increased. CuAl_2_ transforms to Cu_9_Al_4_ over time. During the solidification stage, the residual liquid filler consists of a solid solution (Cu–Al, Ti–Al, Fe–Si) matrix, which acts as a brazing seam. It should be noted that because most of the Zn evaporates in the AlCuSiZnFe HEA during brazing but together with the Ti substrate, the atomic fractions of individual elements excluding Zn in the matrix remain similar while Zn reduces. The composition is similar to that of the HEA system. This type of behavior is also consistent with the results reported by Shun et al.^51^. Therefore, it is inferred that the Ti/AlCuSiZnFe HEA constitutes a robust brazing seam across the joint.

### Joint shear test

**Figure 9:**
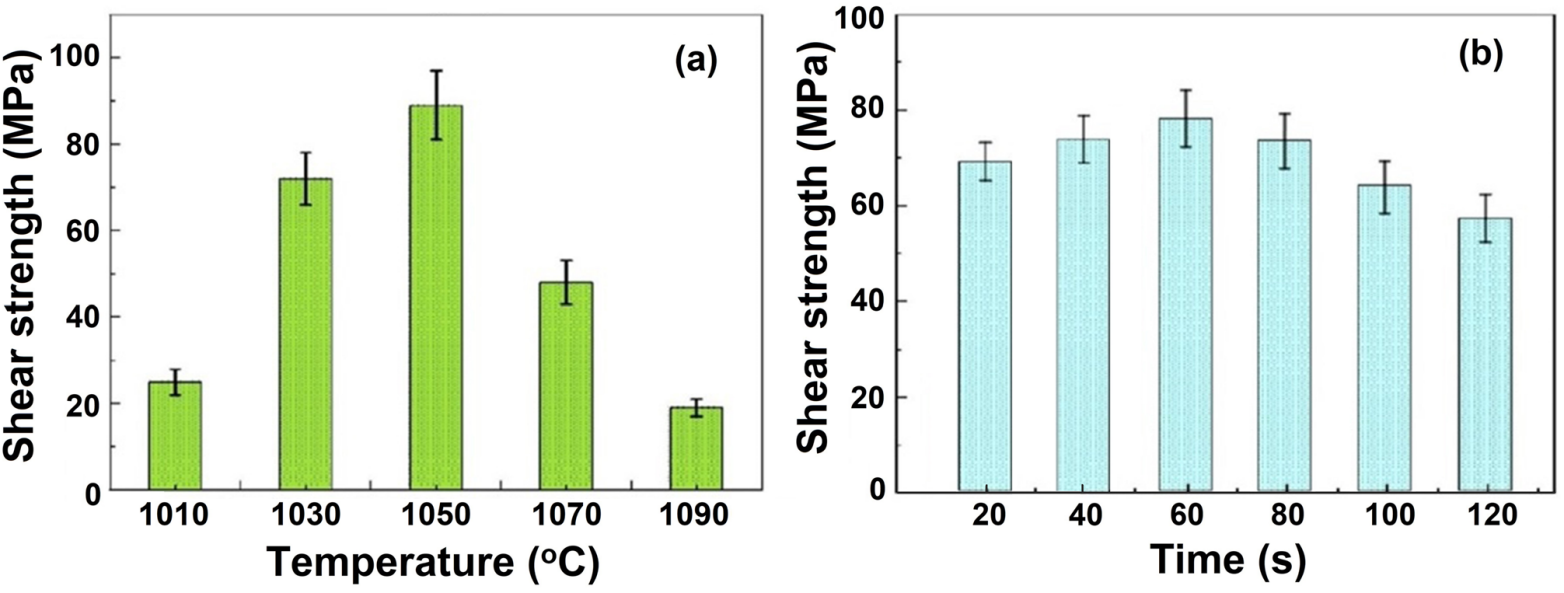
Al_2_O_3_/Ti–6Al–4V joint shear strength at various (**a**) temperatures and (**b**) times.

Figure 9a,b shows the shear strength of the joint at 60 s for various temperatures and at 1050 °C for various holding times. The effects of brazing time and temperature on the shear strength are almost similar. The shear strength increases with temperature which is maximum (84 MPa) at 1050 °C and then reduces. Similarly, the shear strength is the maximum (79 MPa) at a holding time of 60 s at 1050 °C. The shear strength beyond 1050 °C or 60 s decreases owing to the propagation of cracks in the joint. High amount of residual stresses is developed due to the mismatch of coefficients of thermal expansion between Al_2_O_3_ and Ti lowers the joining characteristics^55,56^.

The load-bearing ability of the brazed joints in lap geometry depends upon the width of the reaction layer and heterogeneities (dispersed phase, reinforcement, pores, reaction compounds) present in the brazed joint^57,58^. Enhancement in the shear strength is noted with the increase in the reaction compounds (CuAl_2_, Cu_9_Al_4_, Ti_5_Si_3_) either by increasing the hold time or altering the temperature follows dispersion hardening of the joint. The contribution to strengthening mechanisms can be given by the sum of various strengthening mechanisms operating in the matrix. The dispersed IMCs inside the soft matrix (Cu–Zn) resists the motion of dislocations through dispersion hardening according to the Orowan–Ashby relation^59^:

26 $$\sigma \left(orowan\right)=\frac{0.13Gb}{\lambda }ln\frac{d}{2b}$$

where, G is the shear modulus of Cu, *b* is the Burgers vector, d is the particle size of the IMC phases respectively. The interparticle distance (λ) of the various IMCs with volume fraction *f* in the Cu–Zn matrix can be given by^60^:

27 $$\lambda =\frac{2\left(1-f\right)d}{3f}$$

Accordingly, when $f\to \infty$, $\lambda \to 0$. The decreased spacing between IMCs increases the stress required for the motion of dislocations leading to an increased joint shear strength given by

28 $$\tau =\frac{Gb}{\lambda }$$

The value of G = 45 GPa and *b* = 0.255 nm. For simplicity we assume the IMCs as spheres of diameter 100 nm from the SEM images, λ = 66 nm, *f* = 5 vol.%. The $\sigma \left(orowan\right)$ was caluculated to be 9 MPa.

Considering the grain size strengthening, the Hall–Petch relation is given by^61^:

29 $$\sigma \left(Hall{-}Petch\right): \sigma y=\sigma 0+\frac{k}{{d}^{0.5}}$$

where, *k* is the Hall–Petch constant, $\sigma y, \sigma 0$ are the yield strength of polycrystal and single crystal respectively. Assuming *k* = 0.1 GPa^62^, $\sigma \left(y\right)=7 \mathrm{MPa}.$

Solid solution strengthening was also considered according to the following relation^63^:

30 $$\sigma (solid{-}solution) : {\sigma }_{ss}=\frac{MG{\delta }_{ss}^{3/2}{c}^{1/2}}{700}$$

where *c* is the atomic ratio of solute elements and ${\delta }_{ss}=\left|3{\delta }_{matrix{-}solute}\right|$, M = 3.06 for FCC crystals (highest FCC phase fraction in HEAF, 67% from Table 4). The variable ${\delta }_{matrix-solute}$ is the lattice mismatch between corresponding solute and solvent elements^37,64^. Given the lattice constants of Cu = 0.361 nm and Al = 0.405 nm^65^, the solid solution contribution strengthening is ~ 17 MPa. Similarly, for Cu and Ti, it would be 27 MPa; while for Cu and Zn, it is 68 MPa. The total contribution from all the strengthening mechanisms is around 111 MPa. However, the experimentally observed maximum shear strength is 84 MPa. A similar behavior was recently observed by Li et al.^66^, where the authors studied the Cu/steel joint. According to their research, the discrepancy in the strength values are related to the mechanical constraint imposed by the geometrically necessary dislocations at the brazed joint. In our study, we produced HEAF by milling and spark plasma sintering approach, where dislocations are severely introduced and annihilated at a higher brazing temperature. We see that the strengthening contribution part is significantly higher for Cu–Ti and Cu–Zn solid solutions for Ti/Al_2_O_3_ brazed joint although more studies are needed to generalize these results beyond the binary alloys.

**Figure 10:**
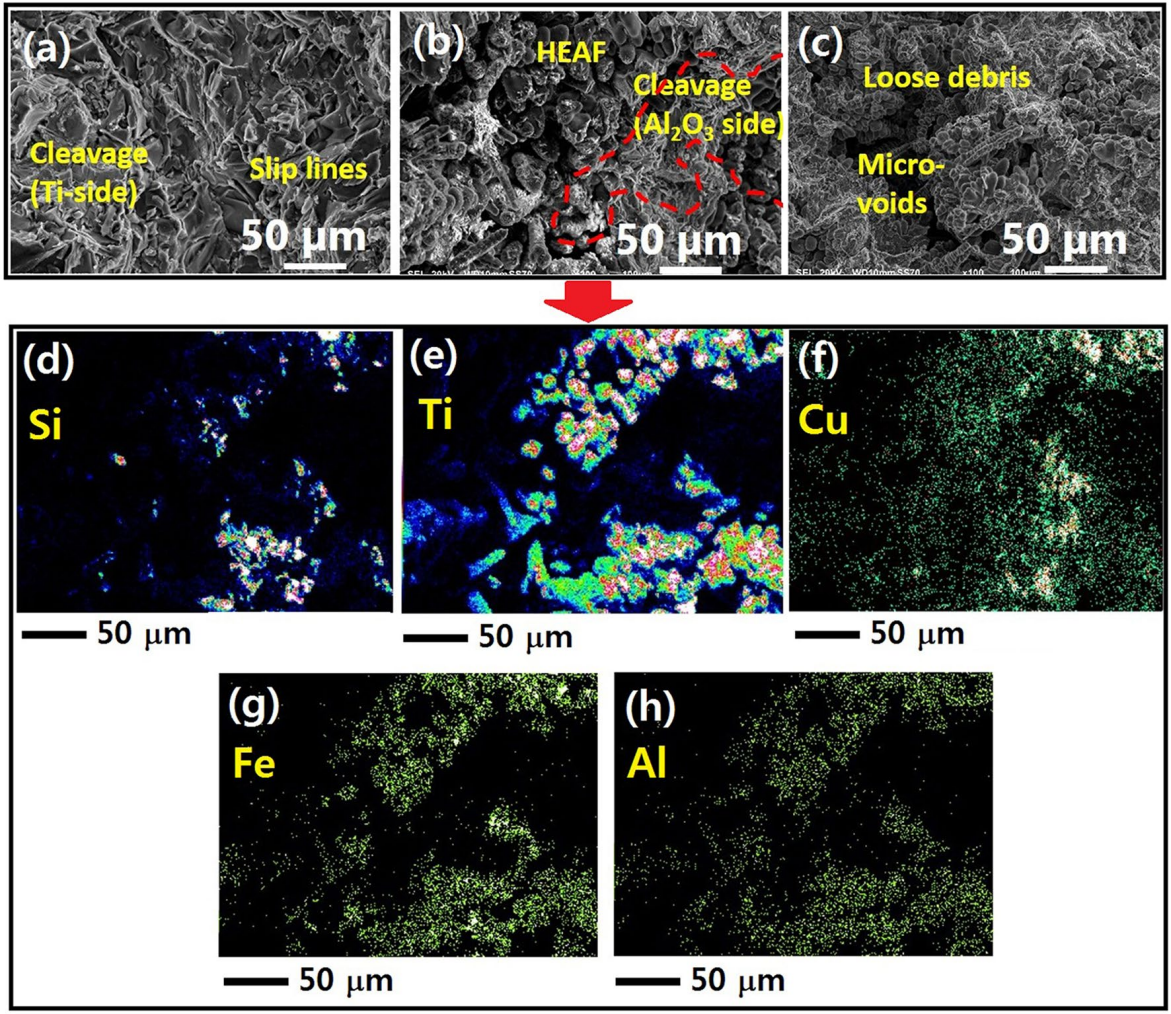
Shear fractured images of the Al_2_O_3_/Ti–6Al–4V joints at (**a**) 1010 °C for 60 s, (**b**) 1050 °C for 60 s, (**c**) 1070 °C for 60 s, and (**d**–**h**) EDS mapping of joint at 1050 °C for 60 s.

As shown in Fig. 10, the fractured surface morphology of the samples confirms the presence of loose debris and mixed cleavage type features (Fig. 10a–c). At 1070 °C, few cleavage steps and a network of slip lines can be seen from the brazing seam on Ti side. The Ti–6Al–4V is produced by a layer-wise additive manufacturing process, therefore, fracture occurs along a preferred direction.

When the temperature increases to 1050 °C, a layered region of Ti–6Al–4V changes to loose debris and sharp cleavage structure arising from Ti–Cu and Ti–Al phases on the Ti side, which induce thermal stresses at the joint causing propagation of cracks and complete failure. However, numerous cracks are present at 1070 °C, and high-resolution images show shallow microvoids mixed with cleavage steps that confirm high thermal stress and quick failure. The thickening of the interface layer creates a wide mismatch in the coefficients of thermal expansion between ceramic and components of the interfacial region. It is inferred that the wide reaction layer deteriorates the joint performance^67^.

## Conclusions

In this study, we developed a novel AlZnCuFeSi HEAF for the microjoining of Al_2_O_3_ and Ti–6Al4V. The important conclusions that can be drawn are summarized below.

1. The use of a high energy ball milling led to the formation of the predominant FCC phase with a minor BCC phase fraction after 45 h of milling. The densification of the powder at 650 °C produced a bulk dual-structured HEAF because of the high positive enthalpy of mixing of the Cu and Zn elements compared to others. The density of the HEAF was ≈ 5 g/cm^3^ with 97–98% densification. The microhardness was approximately 875 HV.
2. The spreading ratio of HEA fillers was the highest for Ti and least for the Al_2_O_3_ substrate. However, irrespective of the spreading conditions, the spreading ratio was greater than 75%, which is reasonable for brazing metals and ceramics. The contact angle improved with an increase in temperature and time being optimal at 1050 °C and 60 s.
3. The use of AlZnCuFeSi HEA as a filler significantly affected the microstructural evolution of the Ti/Al_2_O_3_ joint due to the high-entropy effect. The formation of the joint can be categorized into three stages: Ti/AlCuSiZnFe filler melting, the interfacial dissolution reaction between Ti–Si and CuAl, and the solidification of braze alloy forming Ti–Fe–Si, Ti–Al, and Cu–Al zones in the brazing seam.
4. The brazing temperature has a higher effect on the interface microstructure and width of the Ti/Al_2_O_3_ joint. At low temperatures, the high-entropy effect of the braze alloy maintains the activity of Ti and Zn, while brittle CuAl_2_ IMCs are formed in the brazing seam.
5. The fracture surface of the Al_2_O_3_/Ti–6Al–4V joint shows the presence of cracks at higher temperatures and failure. The maximum joint shear strength is 84 MPa, when brazed at 1050 °C for 60 s. A high brazing temperature leads to the formation of brittle CuAl_2_ IMCs, which become the fracture site of the joint. A smaller brazing time leads to a thinner interface and poor strength, while a prolonged brazing time results in a thick interface zone, which causes quick failure of the joint.

## Data Availability

The data required to reproduce these findings cannot be shared at this time as the research data is confidential.
